# The antioxidant and anti-inflammatory effects of *Eremina desertorum* snail mucin on experimentally induced intestinal inflammation and testicular damage

**DOI:** 10.1042/BSR20221020

**Published:** 2022-10-28

**Authors:** Amina M. Ibrahim, Mostafa Y. Morad, Manal F. El-Khadragy, Olfat A. Hammam

**Affiliations:** 1 Department of Medical Malacology, Theodor Bilharz Research Institute, Giza, Egypt; 2 Department of Zoology and Entomology, Faculty of Science, Helwan University, Egypt; 3 Department of Biology, College of Science, Princess Nourah bint Abdulrahman University, P.O. Box 84428, Riyadh 11671, Saudi Arabia; 4 Department of Pathology, Theodor Bilharz Research Institute, Giza, Egypt

**Keywords:** antioxidant, Eremina desertorum, intestinal inflammation, Mollusca, mucin, testicular damage

## Abstract

*Eremina desertorum* snail mucin antioxidant and anti-inflammatory effects were investigated against carbon tetrachloride (CCl_4_)-intestinal inflammation and testes damage. Male albino mice were intraperitoneally injected with 0.5 ml/kg b.wt of 40% CCl_4_, twice a week for 8 weeks. The treated groups were treated orally with mucin (after 8 weeks of CCl_4_ intoxication, twice a week for 4 weeks). CCl_4_ caused significant increases in C-reactive protein, lipid peroxidation, interleukin-2 levels and caspase-3, while decreasing the total proteins levels, activities of catalase, superoxide dismutase, and glutathione reductase contents, testosterone and 17β estradiol levels compared with the control mice. The improvements of these parameters occurred after treatment with *E. desertorum* mucin, where all the biochemical measurements tended to restore to the normal values. Histopathologically, CCl_4_ caused ulceration in the columnar mucin secreting cells that lined the ileal mucosa, partial loss of goblet cells, abnormal villous/crypt ratio, and submucosal infiltrate of the inflammatory cells. Also, sections of testis showed alterations in the developmental spermatogenic arrangement of the same seminiferous tubules, with no spermatozoa in the center. Improvements in these architectures occurred after administration of mucin, where sections showed almost normal histological structure. In conclusion, *E. desertorum* mucin could be used as a supplementary material as it has antioxidant and anti-inflammatory effects; besides it has low cost.

## Introduction

Phylum Mollusca contained widely distributed invertebrates that inhabited marine, freshwater and terrestrial habitats [1–3]. Many species belong to this Phylum are characterized by having bioactive components that have antioxidants, antibacterial, anticancer, and antiviral activities [4]. Due to the higher protein content of the land snails tissues, they were used as food resources in many countries [5]. Besides being used in folk medicine, their mucous has drained a wide concern nowadays [2,6,7]. The mucous contained a beneficial pharmacological materials that could be used in the biomedical applications like glycosaminoglycan and mucopolysaccharides [8,9]. Recent studies confirmed that the mucous of *Helix aspersa* snails contained natural antioxidant and anti-inflammatory components that ameliorated colon inflammation [10–12]. Mucin is a derivative from mucous that has many advantages and curative effects in wound healing and skin disturbances [10,13] as it could inhibit the inflammatory process due to its protein content [9,13]. Also, it could be used as antioxidant and hepatoprotective agents against CCl_4_-induced liver damage [7]. *Eremina desertorum* (Forsskal, 1775) snails lived in deserts and secreted mucus from their pedal gland [13,14]. GC-MS/MS analyses led to the identification of 10 compounds that were sesquiterpenes, fatty acid esters, monoterpenes, and quinolones [7]. Furthermore, the study of Atta et al. [15] proved that mucin extracted from *E. desertorum* snails’ mucus had promising effects against cancer and oxidative stress damages; thus, it could be used as a natural therapeutic source fighting colon and liver cancers.

Several environmental toxicants could cause severe damages to different organs of the body [16]. Carbon tetrachloride (CCl_4_) is an organic compound used as solvent of oil, in the fumigation of grains, in dry cleaning and as an insecticide [17,18]. The action of CCl_4_ depends on the induction of oxidative stress and causing acute hepatic damage in experimental models [19,20]. It is not only toxic to liver but also to brain, lung, heart, intestine, kidney and testes [21]. The oxidative damage that occurred by CCl_4_ in tissues is due to lipid peroxidation after conversion of CCl_4_ to trichloromethyl radicals, which is highly toxic free radicals [21]. The increased production of CCl_3_ free radicals resulted in the increase of lipid and protein oxidation leading to many pathological alterations [22]. These consequences lead to a great inflammatory effect on intestinal mucosa where CCl_4_ inhibited protein synthesis when added *in vitro* and due to increased lipid peroxidation [23]. Testes have a great affinity to accumulate CCl_4_ and this could lead to severe damages in it [20]. CCl_4_ resulted in the changes in the testes architecture where it increased the sperm shape abnormality and sperm DNA tail moment [22,24].

Therefore, the current study aimed to investigate the ameliorative effects of *E. desertorum* snails’ mucin on experimentally induced intestinal inflammation and testes damages by CCl_4_, through studying different biochemical, histopathological parameters.

## Materials and methods

### Preparation of the *E. desertorum* slime and mucin

To extract the slime, we used the simplest means in heliciculture, and this allowed us to have a pure fresh slime. Briefly, a sterile wooden rod was used to stimulate the snail, by rubbing its muscular foot with rod, enhancing the snail to secret more slime. The slime collected was kept in a sterile container and then preserved at (−30°C) until further use.

In a 40°C water bath, the slime was macerated for 24 h. The process of mixing the water twice the number of samples added to the slime yielded a fraction containing water-soluble slime. The supernatant was received as WSF (water-soluble fraction). The WSF slime fraction (mucin fraction) was obtained by ethanol precipitation, which involved mixing the supernatant from the water maceration with an absolute ethanol ratio of 1:3 and centrifuging it for 30 min at 2900 × ***g***. After re-dissolving the precipitate (5 g) in Tris-HCl, the mucin fraction was recovered [9].

### Animals

White male albino mice of CD1, aged 6–8 weeks and weighing (18–20 g), were obtained from the Animal House from the Schistosome Biological Supply unit, Theodore Bilharz Research institute, Giza-Egypt (SBSP, TBRI). Mice were maintained for 2 weeks in plastic cages in an animal room, at temperature ranging between 20 and 25°C and were fed Purina chaw (20% protein) and given tap water. All ethics were approved by the Ethics Committee of Theodor Bilharz Research Institute (TBRI) number [PT (511)]. All animal experiments took place at Bilharz Research Institute.

### Experimental design protocols

The mice were randomly divided into three groups (20 mice each) as follows:

- Negative control group: 20 mice were injected intraperitoneally with 0.5 ml/kg b.wt of sterile olive oil twice a week for 8 weeks.
- Positive control group: 20 mice were injected intraperitoneally with 0.5 ml/kg b.wt of 40% CCl_4_ (a mixture of pure CCl_4_ obtained from Adwic Chemicals Co. (Cairo-Egypt) and sterile olive oil v/v), twice a week for 8 weeks.
- CCl_4_+Mucin group: 20 mice were orally administered, after 8 weeks, 20 ml of mucin/kg, twice a week for 4 weeks.

After the end of the experimental period (12 weeks), all animals were killed by cervical dislocation and blood samples were collected.

### Serum preparation and biochemical investigation

Blood was allowed to stand at 37°C for 1 h, then over night at 4°C, and centrifuged at 3000 × ***g*** for 30 min. Sera were separated and heat-inactivated at 56°C for 30 min and stored in aliquots at −20°C, until use. After 15 days of the experiment, all animals were anesthetized with chloroform for 2 min before withdrawal of blood samples; a capillary was inserted in the cavernous sinus of the animal and blood obtained was directly collected in dry tubes for the analyses of C-reactive proteins (CRP). All tubes undergone centrifugation at 5000 × ***g*** for 10 min. The supernatants were collected and conserved at –30°C until use. CRP serum levels were measured by an immunoturbidimetric method using commercial Randox kit (U.K.) with standards [10]. Total protein concentration was determined in liver homogenate and serum according to the method of Doumas [25].

### Investigation of the oxidative stress biomarkers

The supernatant of the tissue homogenate for each group was used to investigate the oxidative stress enzymes. To determine superoxide dismutase (SOD) and catalase (CAT) concentrations, bio diagnostic kits (Biodiagnostic, Giza, Egypt) were used. The tissue malondialdehyde (lipid peroxide) activity was estimated according to Ohkawa et al. [26], and by using the method of Ellman [27], reduced glutathione (GSH) was evaluated.

### Enzyme-linked immunosorbent assay (ELISA)

Serum murine IL-2 levels were detected using ELISA kit (SinoGeneClon Biotech Co., Ltd) according to Engvall and Perlmann [28]. The cytokine concentration was obtained from a regression curve prepared with the help of microplate manger software (Bio-Rad).

Caspase-3 activity was determined according to Bonomini et al. [29]. The released p-nitroaniline (pNA) moiety concentration was measured colorimetrically at 405 nm.

### Investigation of testosterone and 17β estradiol hormones in serum

Testosterone hormone activity was determined in serum of all groups according to the manufacturer instructions of testosterone ELISA kit (Enzo Life Science, Michigan, U.S.A., ADI-900-065) while 17β estradiol was analyzed according to ELISA kit (Cayman Chemical Company, Michigan, U.S.A., item no. 582251).

### Histopathological studies

Ileum and testes tissues were isolated from each group, washed with saline and fixed in 10% buffered formalin. Fixed samples were dehydrated in ascending series of alcohol, then cleaned with xylene and embedded in paraffin. Sections of the tissues 5 μm thickness were prepared and stained with hematoxylin and eosin (H&E).

### Statistical analysis

The data were represented as mean ± S.D and by using Student’s *t*-test, the comparison between two means was conducted. The *P* value less than 0.05 was considered as statistically significant. The data analysis was done with SPSS version 20.

## Results

**Figure 1:**
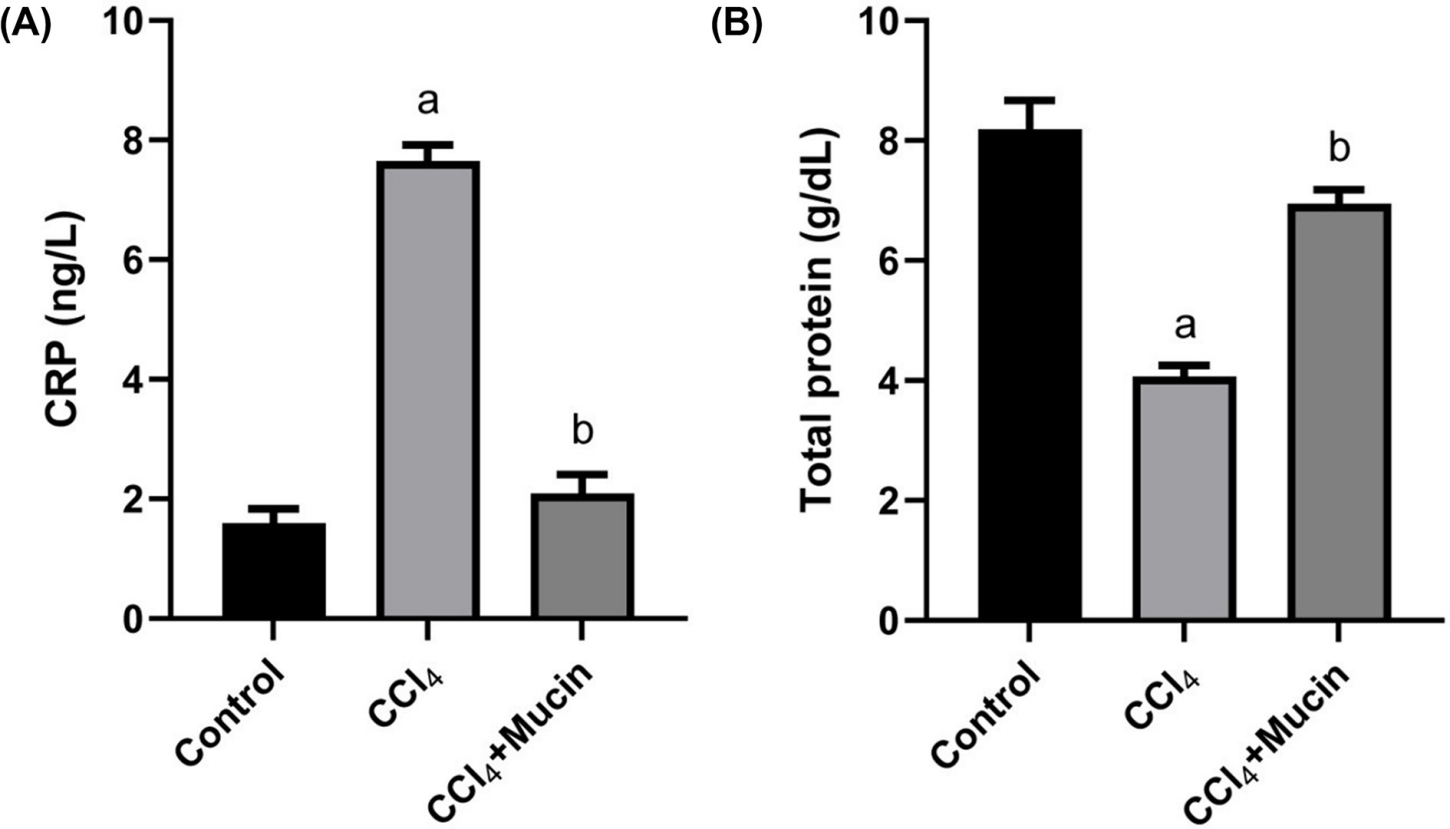
Effect of CCl4 and* E. desertorum* snail mucin treatment on CRP and total protein Effect of CCl_4_ and *E. desertorum* snail mucin treatment on (**A**) the CRP and (**B**) total protein of treated mice. Data are presented as means and standard deviation. Significant differences between control and treated mice are indicated with a at *P*<0.05. While, significant differences between CCl_4_ and CCl_4_+Mucin treated mice are indicated with b at *P*<0.05.

The current results showed significant increases (*P*<0.01) in CRP accompanied with a significant decrease in the total proteins levels in CCl_4_ intoxicated group compared with those of the normal group. While, mice received CCl_4_ and *E. desertorum* snail mucin showed significant improvements in these parameters compared with the group received CCl_4_ alone (Figure 1A,B).

**Figure 2:**
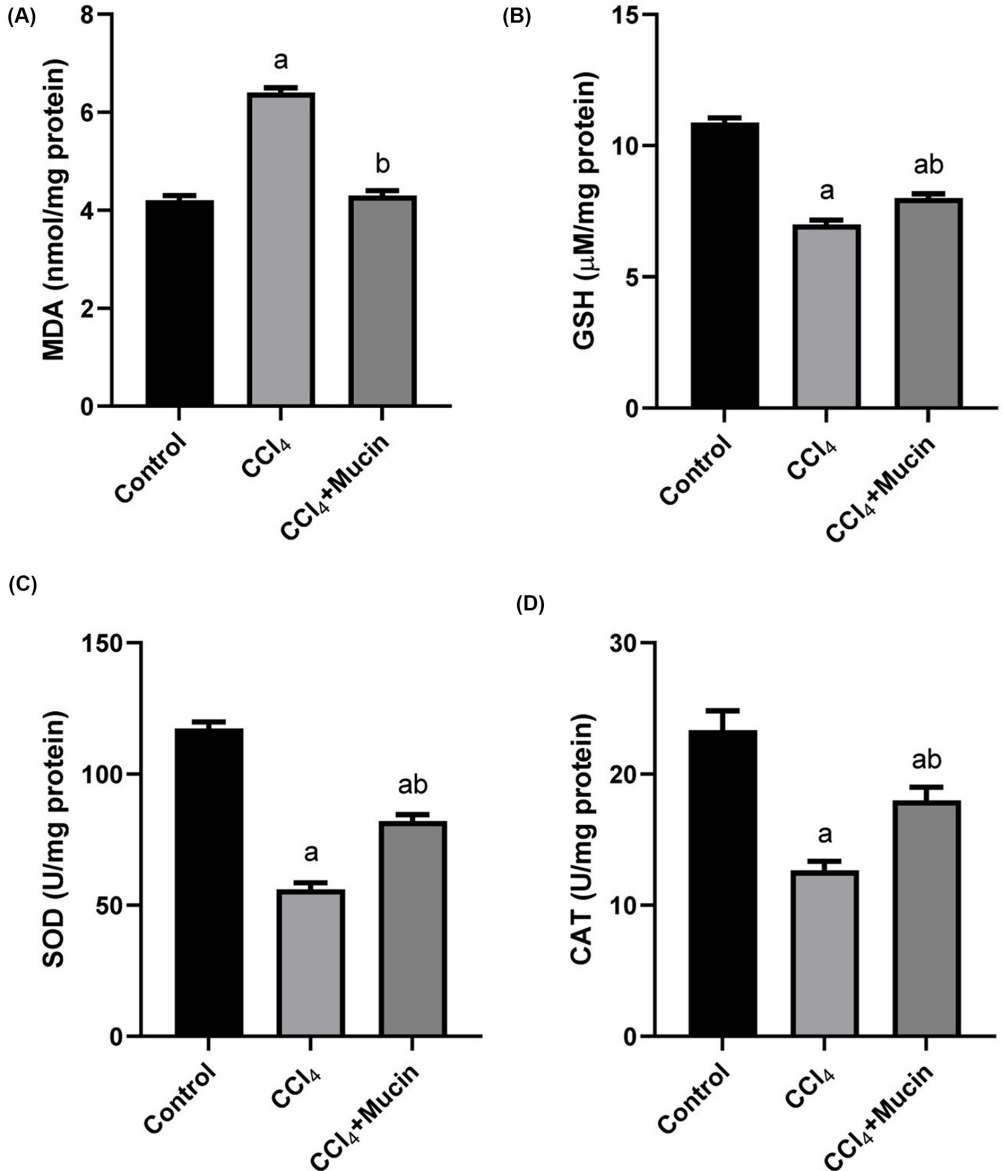
Effect of CCl_4_ and *E. desertorum* snail mucin treatment on malondialdehyde and antioxidant enzymes Histogram shows the effect of CCl_4_ and *E. desertorum* snail mucin treatment on the mean levels of (**A**) malondialdehyde (MDA); (**B**) reduced glutathione (GSH); (**C**) the mean activities of superoxide dismutase (SOD), and (**D**) catalase (CAT) enzymes of treated mice. Data are presented as means and standard deviation. Significant differences between control and treated mice are indicated with a at *P*< 0.05. While, significant differences between CCl_4_ and CCl_4_+Mucin treated mice are indicated with b at *P*<0.05.

Intoxication of mice with CCl_4_ significantly increased (*P*<0.01) MDA levels, while decreased the activities of CAT and SOD and GSH contents compared with control normal group. On contrast, the administration of *E. desertorum* snail mucin resulted in significant improvements in all of these parameters compared with CCl_4_ intoxicated group to restore the normal readings (Figure 2A–D).

**Figure 3:**
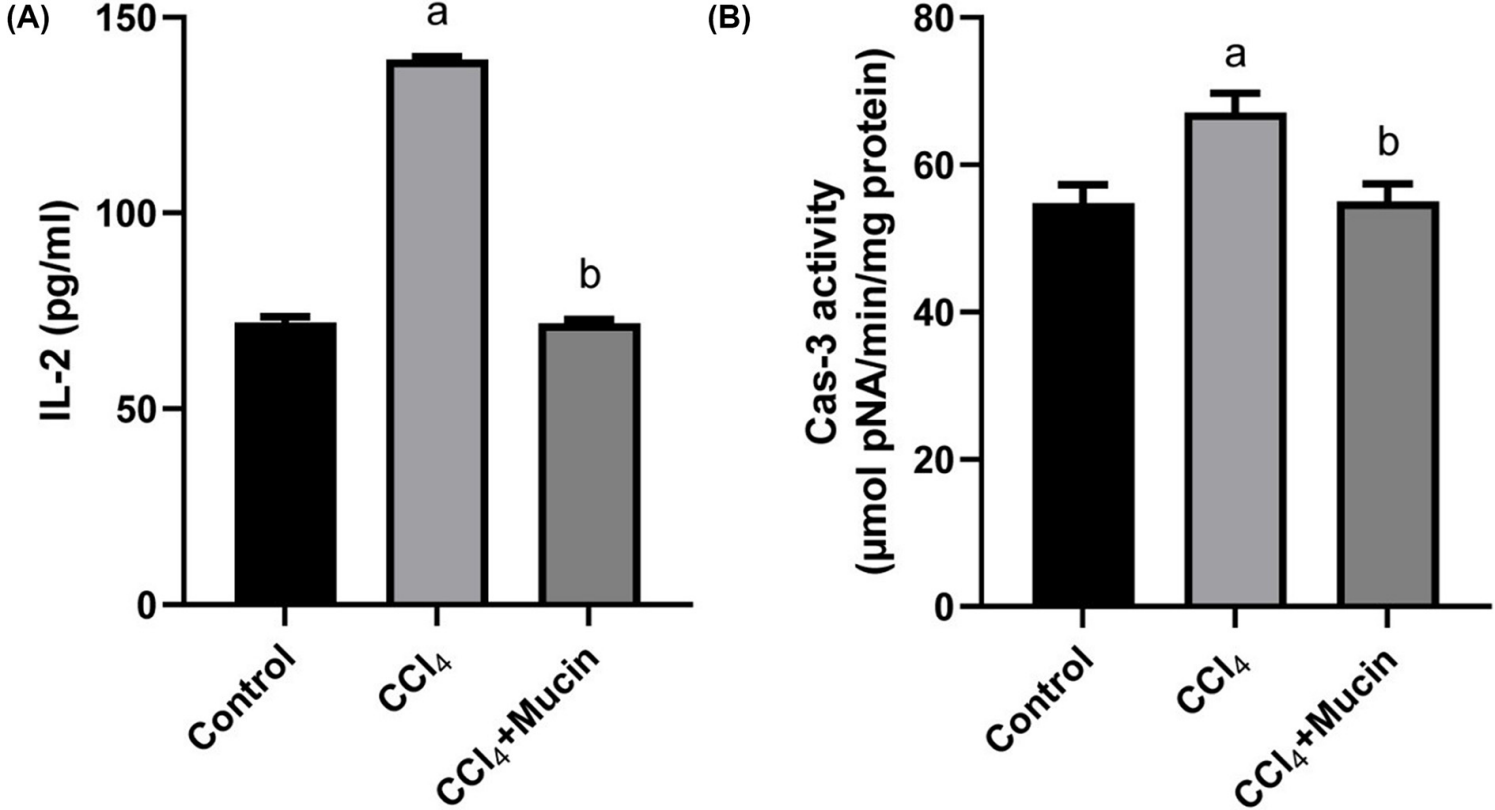
Effect of CCl_4_ and *E. desertorum* snail mucin treatment on IL-2 level & caspase-3 activities shows the effect of CCl_4_ and *E. desertorum* snail mucin treatment on (**A**) IL-2 level and (**B**) caspase-3 activity of treated mice. Data are presented as means and standard deviation. Significant differences between control and treated mice are indicated with a at *P*<0.05. While, significant differences between CCl_4_ and CCl_4_+Mucin treated mice are indicated with b at *P*< 0.05.

The present investigation showed that mice induced with CCl_4_ has significant (*P*<0.05) increase in IL-2 level and caspase-3 activity compared with control group. On contrast, the administration of *E. desertorum* mucin resulted in significant improvement (*P*<0.05) in IL-2 level and caspase-3 activity compared with those of mice administered CCl_4_ alone (Figure 3A,B).

**Figure 4:**
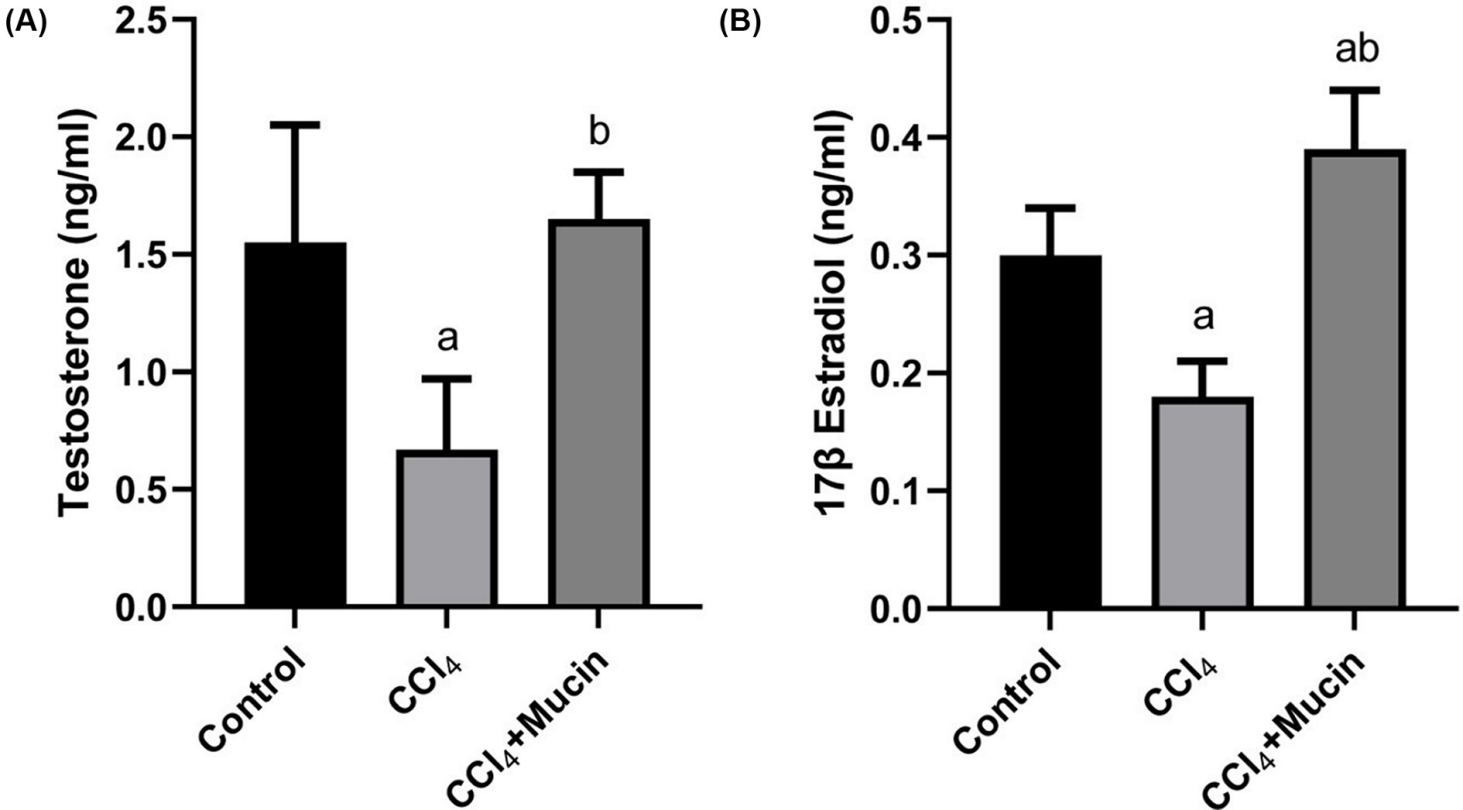
Effect of CCl_4_ and *E. desertorum* snail mucin treatment on Testosterone and 17β Estradiol Histogramshows the effect of CCl_4_ and *E. desertorum* snail mucin treatment on (**A**) Testosterone (T) and (**B**) 17β Estradiol (E). Data are presented as means and standard deviation. Significant differences between control and treated mice are indicated with a at *P*<0.05. While significant differences between CCl_4_ and CCl_4_+Mucin treated mice are indicated with b at *P*< 0.05.

The present results revealed that CCl_4_ intoxicated mice had significant decreased (*P*<0.05) in testosterone (T) and 17β estradiol (E) compared with control group. While the administration of *E. desertorum* mucin caused significant increase (*P*<0.05) in both T and E compared with intoxicated group (Figure 4A,B).

Histopathogical sections of ileum after intoxication with CCl_4_ showed that there was ulceration in the mucin secreting cells with partial loss of goblet cells, atrophic mucosa (with abnormal villous/crypt ratio) and submucosal infiltrate of inflammatory cells. While mucin treated group showed ileal mucosa lined by columnar mucin secreting cells with mild broad and blunt villous and mild depletion of goblet cells normal mucosa (with normal villous /crypt ratio), and submucosa and villous core are infiltrated with mild number of lymphocytes, muscle layer (Figure 6A–C).

**Figure 5:**
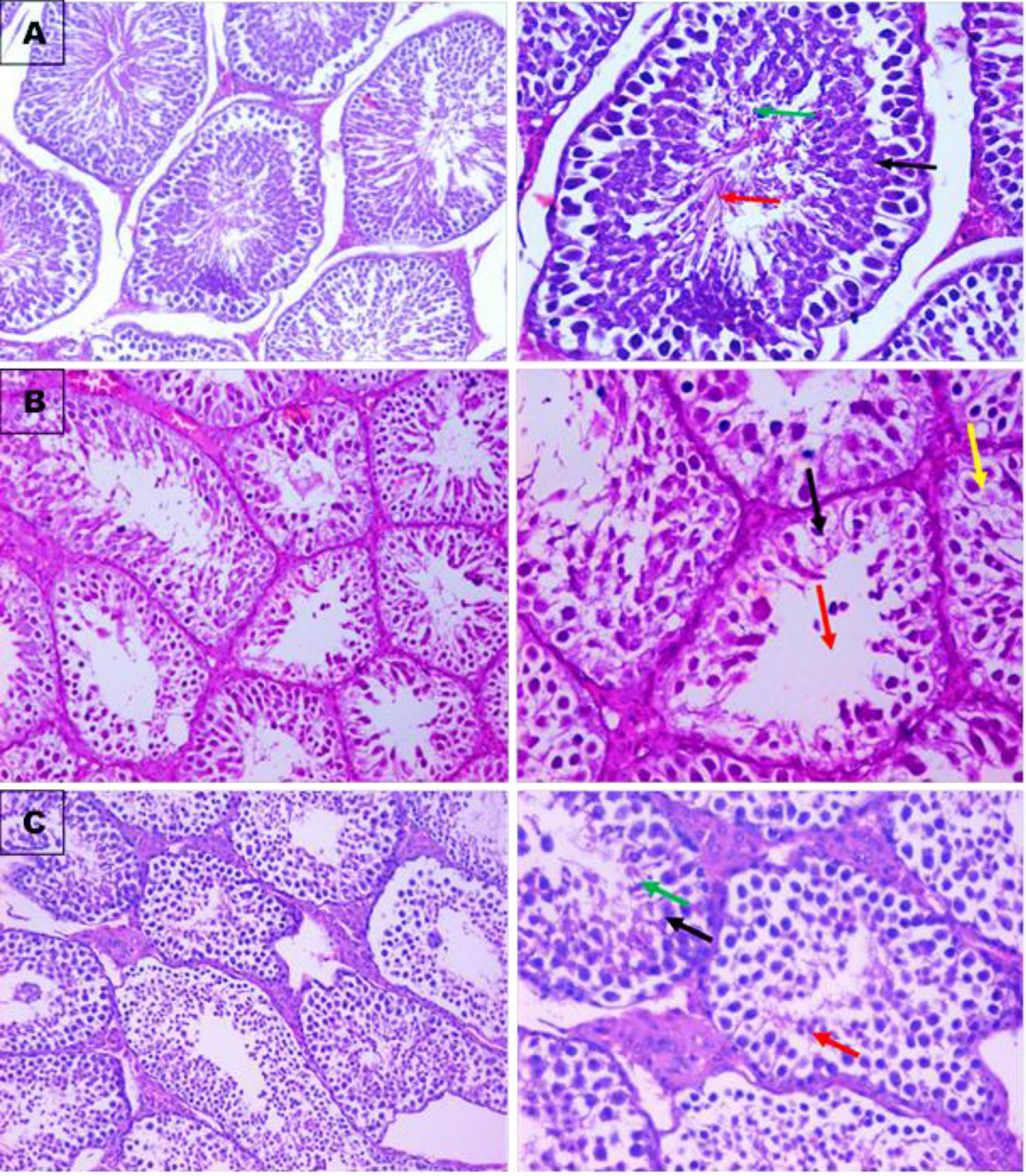
Photomicrographs for testis sections stained with hematoxylin and eosin (H&E) Section of testis showing: (**A**) normal control group with normal histopathological structure of mature active seminiferous tubules with complete spermatogenic series, primary spermatocyte (black arrows), spermatid (green arrow) with large number spermatozoa in the center (red arrow). (**B**) Intoxicated CCl_4_ group with degeneration and lose of spermatogenic series in some of the same seminiferous tubules (black arrow), with no of spermatozoa in the center (red arrow), few normal seminiferous tubules (yellow arrow) with incomplete spermatogenic series. (**C**) Mucin treated group showing mostly almost normal histopathological structure of mature active seminiferous tubules with complete spermatogenic series, primary spermatocyte (black arrows), spermatid (green arrow) with moderate number of spermatozoa in the center (red arrow). H&E, ×200 right side, ×400 left side.

**Figure 6:**
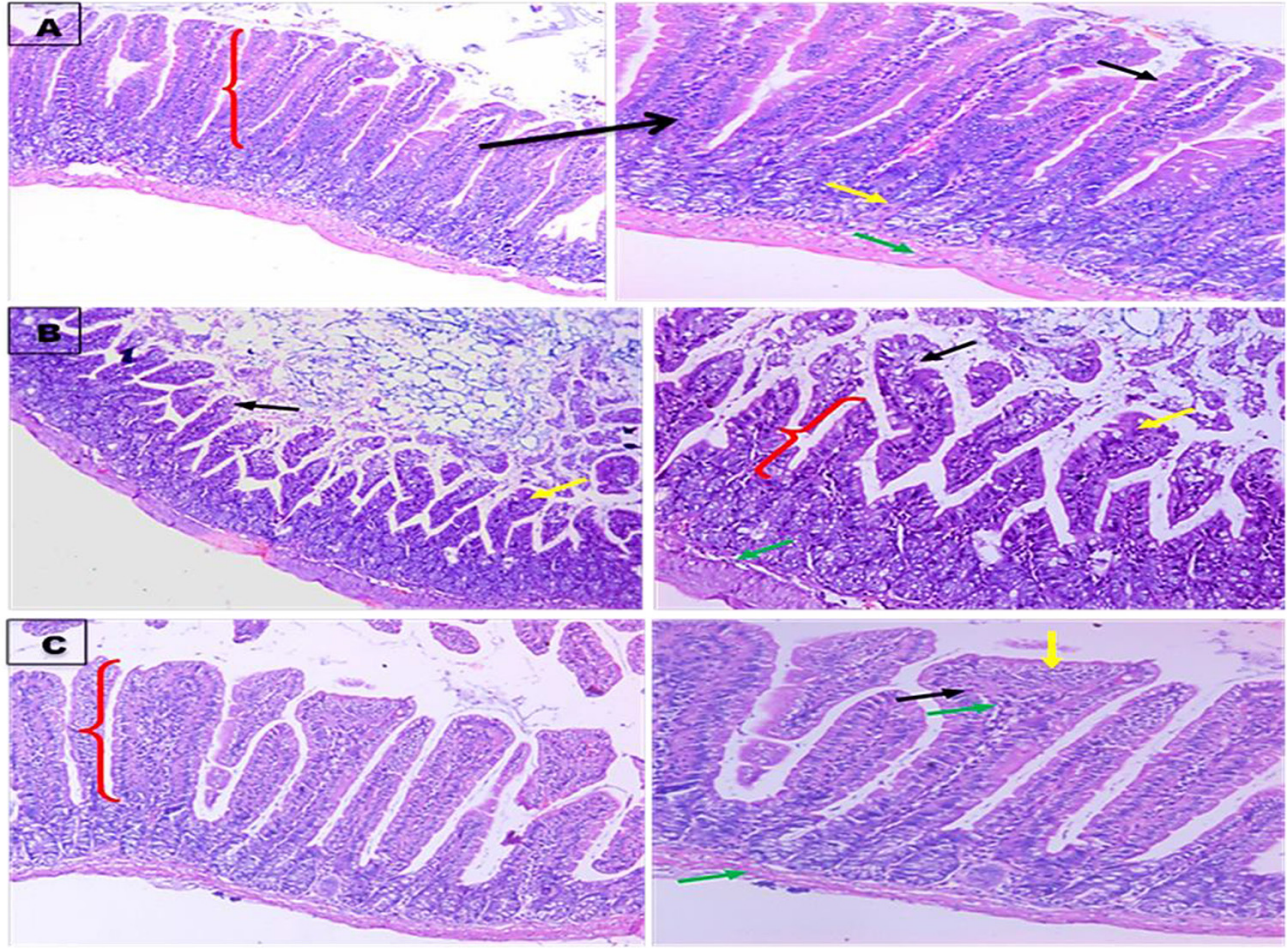
Photomicrographs for ileum small intestine sections stained with hematoxylin and eosin (H&E) Histological sections of ileum small intestine. (**A**) normal control group showing ileal mucosa lined by columnar mucin secreting cells with normal villous pattern and goblet cells (black arrow) normal mucosa (with normal villous/crypt ratio) (red arrow), and sub mucosa (yellow arrow), muscle layer (green arrow). (**B**) (+ve control), showing ileal mucosa lined by columnar mucin secreting cells with ulceration (black arrow), short blunting of villi and broad villi (yellow arrow), with partial loss of goblet cells, atrophic mucosal mucosa (with abnormal villous/crypt ratio) (red arrow) and submucosal (green arrow) infiltrate of inflammatory cells. (**C**) Mucin treated group showing ileal mucosa lined by columnar mucin secreting cells with mild broad and blunt villous (yellow arrow) and mild depletion of goblet cells (black arrow) normal mucosa (with normal villous /crypt ratio) (red arrow), and submucosa and villous core are infiltrated with mild number of lymphocytes (green arrow), muscle layer (green arrow); H&E, ×100 right side, ×200 left side.

Also, the present histopathological sections of testes showed that intoxication with CCl caused degeneration with lose of spermatogenic series in some of the same seminiferous tubules, with no of spermatozoa in the center, few normal seminiferous tubules with incomplete spermatogenic series (Figure 5B). While section of testis from mucin treated group restored its normal histopathological nature of mature active seminiferous tubules with complete spermatogenic stages, primary spermatocyte, spermatid with moderate number of spermatozoa in the center (Figure 5C).

## Discussion

Supplementary medicaments of natural origin could decrease the oxidative stress occurred in the damaged tissue [30]. In a previous study, carried out by Ibrahim et al. [7], the GC-MS analysis of mucin extracted from mucus of *E. desertorum* snails confirmed that it had many active bio-components like benzo[f]quinoline, cyclopentasiloxane, decamethyl, glycerol 1,2-diacetate, hexadecanoic acid, ethyl ester, tert-butyldimethylsilyl ester; and thiophene. In addition, Sallam et al. [31] stated that GC-MS analysis of the mucus of *Theba pisana*, *Eobania vermiculata* and *Monacha obstructa* snails, showed the presence of Oxime, methoxy-phenyl and cyclotrisiloxane, hexamethyl as major components in their mucous. These materials have antioxidant and anti-inflammatory activities [10,14]. Moreover, Ibrahim et al. [7] revealed that *E. desertorum* mucin could be used as a potential antioxidant, hepatoprotective and anti-inflammatory agent for hepatic disorders against CCl_4_ induced hepatotoxicity.

Formerly, the slime of *Helix aspersa* snails was confirmed to ameliorate colon inflammation as it contained anti-inflammatory and antioxidant components [10]. Recently, El-Zawawy and Mona [32] confirmed that mucous extracts from *E. desertorum* snails have higher biological effects compared with *H. aspersa*, which recommended to be used for human therapies.

CRP is a protein that produced in the site of the inflammation by hepatocytes and considered as a good biomarker of measuring the inflammation [33]. The present results showed that there was a significant increase in CRP accompanied with a significant decrease in the total proteins levels in CCl_4_ intoxicated group compared with those of the normal group. While the group received CCl_4_ and mucin showed significant improvements in these parameters compared with the group received CCl_4_ alone and tried to restore the normal level. These results in a good accordance with [10] who revealed a significant decrease of CPR in group took slime of *H. aspersa* and acetic acid compared with acetic acid group. They suggested that the slime might have anti-inflammatory properties. Also, the reduction in total protein concentrations after CCl_4_ intoxication might be due to liver damage through induction of lipids peroxidation and cellular membrane inflammation [34].

The antioxidant markers included enzymatic (GST, SOD and CAT) and non-enzymatic (GSH) markers played a vital role in protection the organisms from oxidative stress and suppression of its cellular damage [35]. GSH is a non-enzymatic free radical quencher acting as a substrate for the antioxidant enzyme, glutathione transferase (GST) [20]. The induction of SOD/CAT system could be a first line defense against the overproduction of reactive oxygen species inside the tissue due to the increase of lipid peroxide MDA [19]. The present investigation showed that the intoxication of mice with CCl_4_ significantly increased MDA level, while decreased the activities of CAT and SOD and GSH contents compared with control group. On contrast, the administration of *E. desertorum* snail mucin resulted in significant improvements in all of these parameters compared with CCl_4_ intoxicated group to restore the normal levels. These results were in consistence with previous study of Safhi [36] who reported that that CCl_4_ could reduce CAT, GSH, GST and SOD activities in mice compared to normal group and confirmed that zingerone had ameliorated effects on CCl_4_ induced nephrotoxicity through increasing the antioxidant enzymes than CCl_4_ treated group.

The present investigation showed that mice with CCl_4_ had significant increase in IL-2 level and caspase-3 activity compared with control group. On contrast, the administration of *E. desertorum* mucin resulted in significant improvement in IL-2 level and caspase-3 activity compared with those of mice administered CCl4 alone. Wang et al. [37] correlated the hepato-protective effects of zerumbone to the down-regulating the inflammatory response through the decrease in the production of inflammatory cytokines TNF-α and IL-6 in CCl_4_-intoxication mice. Also, Safhi [36] reported that CCl_4_ increased the cytokines such as IL-1β, IL-2 and TNF-α levels as compared to normal group, while after the treatment with zingerone significantly decreased inflammatory cytokines.

Caspase-3 is a marker of cell death protease and plays a vital role in apoptosis, therefore its overexpression is a sign of great damage in the tissue [20]. Abdel Moneim [20] showed an increase in caspase-3 positive cells in the testes of rats intoxicated with CCl_4_ and related this increase with the necrosis in the testes, oxidative stress and increases apoptosis. However, *Physalis peruviana* fruit could protect testes against CCl_4_-induced apoptosis by inhibiting the expression level of caspase-3.

Carbon tetrachloride could damage all organs of the body; the central nervous system, the liver, kidneys, intestine and testes. So, it might affect pituitary hormone secretions [24]. The present results revealed that CCl_4_ intoxicated mice had a significant decrease in testosterone and 17β estradiol compared with control group. While the administration of *E. desertorum* mucin caused a significant increase in both T and E compared with intoxicated group and tried to restore the normal values. These results were in good accordance of Abdel Moneim [20] who showed that CCl_4_ administration caused testicular atrophy, decrease in testosterone and gonadotropins (FSH and LH) in male rat, and stated that *P. peruviana* fruit could increase testosterone, FSH and LH levels through direct effects on the central nervous system and gonadal tissues or their effects on hypothalamus–pituitary–testis axis.

Untreated small intestine sections showed normal ileal mucosa lined by columnar mucin secreting cells with normal villous pattern, sub mucosa, goblet cells and muscle layer. After intoxication with CCl_4_, the ileal mucosa lined by columnar mucin secreting cells showed ulceration, short blunting and broad villi, with partial loss of goblet cells, with abnormal villous/crypt ratio and submucosal infiltrate of inflammatory cells. Amelioration of this architecture after administration of *E. desertorum* mucin which revealed that ileal mucosa lined by columnar mucin secreting cells with mild broad and blunt villous, mild depletion of goblet cells, with normal villous/crypt ratio, and submucosa and villous core are infiltrated with mild number of lymphocytes and muscle layer. Also, section of testis from control group showed normal histopathological structure of mature active seminiferous tubules with complete spermatogenic series, primary spermatocyte, spermatid and with large number of spermatozoa in the center. Section of testis from CCl_4_ intoxicated group showed degeneration with lose of spermatogenic series in some of the same seminiferous tubules, without spermatozoa in the center, few normal seminiferous tubules with incomplete spermatogenic series. While group treated with *E. desertorum* mucin showed almost normal histopathological structure of mature active seminiferous tubules with complete spermatogenic series, primary spermatocyte, spermatid with moderate number of spermatozoa in the center and almost normal structure and architecture. Unsal et al. [34] stated that brain, lung, heart, liver, the kidney, testes and other tissues showed different deleterious histopathological effects after intoxication with CCl_4_. Foaud et al. [24] reported that N-acetyl cysteine administrations could improve testes, liver and kidney architecture. Also, Sonmez et al. [22] used quercetin to improve CCl_4_-induced sperm damages, testicular apoptosis and oxidative stress in male rats and concluded that quercetin has antiperoxidative effect and could decrease the CCl_4_-induceddamages in male reproductive organs and cells by decreasing lipid peroxidation.

Wand et al. [38] revealed the damages of CCl_4_ on the intestinal mucosa and correlated this damage with the damage of liver. They reasoned these damages to the metabolism of CCl_4_ and lipid peroxidation. Also, many researches confirmed the antioxidant and the anticancer activities of *E. desertorum* snails’ mucin and concluded that it could be used as natural therapeutic agents against colon and liver cancers [10,32].

## Conclusion

The present work revealed that *E. desertorum* mucin could be used as potential antioxidant, anti-inflammatory agents. Further studies are needed to configure the best way to use it as a supplementary drug with low cost and safe to consumer.

## Data Availability

All relevant data are within the paper.

## Abbreviations

CAT: catalase
CCl_4_: carbon tetrachloride
CRP: C-reactive proteins
GSH: glutathione
GST: glutathione transferase
MDA: malondialdehyde
pNA: p-nitroaniline
SOD: superoxide dismutase
WSF: water-soluble fraction

## References

[1] Ibrahim A.M., Hamed A.A. and Ghareeb M.A. (2021) Marine, freshwater, and terrestrial snails as models in the biomedical applications. Egypt J. Aquat. Biol. Fish. 25, 23–38 10.21608/ejabf.2021.172142

[2] Abd-El H., Osman G.Y., El-Sabbagh S.M. and Sheir S. (2020) Antibacterial activity of some terrestrial gastropods from Egypt against Staphylococcus aureus and Escherichia coli. Egy. J. Zoo. 72, 1–12 10.21608/ejz.2020.26009.1024

[3] Neubauer T.A., Harzhauser M., Georgopoulou E., Kroh A. and Mandic O. (2015) Tectonics, climate, and the rise and demise of continental aquatic species richness hotspots. Proc. Natl. Acad. Sci. U.S.A. 112, 11478–11483 10.1073/pnas.1503992112 26305934 PMC4577204

[4] Vinarski M.V., Bolotov I.N., Aksenova O.V., Babushkin E.S., Bespalaya Y.V., Makhrov A.A. et al. (2021) Freshwater Mollusca of the Circumpolar Arctic: a review on their taxonomy, diversity and biogeography. Hydrobiologia 848, 2891–2918 10.1007/s10750-020-04270-6

[5] Nantarat N., Tragoolpua Y. and Gunama P. (2019) Antibacterial activity of the Mucus Extract from the Giant African Snail (Lissachatina fulica) and Golden Apple Snail (Pomacea canaliculata) against pathogenic bacteria causing skin diseases. Trop. Nat. Hist. 19, 103–112

[6] Ulagesan S. and Kim H.J. (2018) Antibacterial and antifungal activities of proteins extracted from seven different snails. Appl. Sci. 8, 1362 10.3390/app8081362

[7] Ibrahim A.M., Hussein T.M., Abdel-Tawab H., Hammam O.A. and Ghareeb M.A. (2022) The ameliorative effects of Eremina desertorum snail mucin in combination with Silymarin against experimentally induced liver fibrosis. Egy. J. Chem. 65, 181–190

[8] Skingsley D., White A. and Weston A. (2000) Analysis of Pulmonate mucus by infrared spectroscopy. J. Moll. Stud. 66, 363–372 10.1093/mollus/66.3.363

[9] Harti A.S., Sulisetyawati S.D., Murharyati A., Oktariani M. and Wijayanti I.B. (2016) The effectiveness of snail slime and chitosan in wound healing. Int. J. Pharma. Med. Biol. Sci. 5, 76–80

[10] Hatuikulipi T., Naimi D., Kouachi M. and Bouchetob L. (2016) Preventive effect of Helix aspersa slime against experimentally chemo-induced colitis in rat. Der. Pharmacia. Lett. 8, 200–206

[11] Trapella C., Rizzo R., Gallo S., Alogna A., Bortolotti D., Casciano F. et al. (2018) HelixComplex snail mucus exhibits pro-survival, proliferative and pro-migration effects on mammalian fibroblasts. Sci. Rep. 8, 17665 10.1038/s41598-018-35816-3 30518946 PMC6281574

[12] Gentili V., Bortolotti D., Benedusi M., Alogna A., Fantinati A., Guiotto A. et al. (2020) HelixComplex snail mucus as a potential technology against O3 induced skin damage. PLoS ONE 15, e0229613 10.1371/journal.pone.0229613 32084249 PMC7034816

[13] Ali M.T., Ashari M.F., Wijaya S.P.R., Lestari E. and Wijayanti R. (2018) Evaluation of wound healing effect of eel mucus ointment (Belutidine) in mice by incision model. J. Nat. Remedies 18, 1–9 10.18311/jnr/2018/18107

[14] Gabriel U.I., Mirela S. and Ionel J. (2011) Quantification of mucoproteins (glycoproteins) from snails mucus, Helix aspersa and Helix pomatia. J. Agroaliment Process Technol. 17, 410–413

[15] Atta S.A., Ibrahim A.M. and Megahed F.A.K. (2021) In-vitro anticancer and antioxidant activities of Eremina desertorum (Forsskal, 1775) Snail Mucin. Asian Pac. J. Cancer Prev. 22, 3467–3474 10.31557/APJCP.2021.22.11.3467 34837901 PMC9068163

[16] Al Olayan E.M., Aloufi A.S., AlAmri O.D., El-Habit O.H. and Abdel Moneim A.E. (2020) Protocatechuic acid mitigates cadmium-induced neurotoxicity in rats: Role of oxidative stress, inflammation and apoptosis. Sci. Total Environ. 723, 137969 10.1016/j.scitotenv.2020.137969 32392679

[17] Peng X., Dai C., Liu Q., Li J. and Qiu J. (2018) Curcumin attenuates on carbon tetrachloride-induced acute liver injury in mice via modulation of the Nrf2/HO-1 and TGF-β1/Smad3 pathway. Molecules 23, 215 10.3390/molecules23010215 PMC6017508 29351226

[18] Hamed S.S., Al-Yhya N.A., El-Khadragy M.F., Al-Olayan E.M., Alajmi R.A., Hassan Z.K. et al. (2016) The protective properties of the strawberry (fragaria ananassa) against carbon tetrachloride-induced hepatotoxicity in rats mediated by anti-apoptotic and upregulation of antioxidant genes expression effects. Front Physiol. 7, 325 10.3389/fphys.2016.00325 27547187 PMC4974471

[19] Elsawy H., Badr G.M., Sedky A., Abdallah B.M., Alzahrani A.M. and Abdel-Moneim A.M. (2019) Rutin ameliorates carbon tetrachloride (CCl4)-induced hepatorenal toxicity and hypogonadism in male rats. PeerJ. 7, e7011 10.7717/peerj.7011 31179192 PMC6545103

[20] Abdel Moneim A.E. (2014) Prevention of carbon tetrachloride (CCl4)-induced toxicity in testes of rats treated with Physalis peruviana L. fruit. Toxicol. Ind. Health 32, 1064–73 25147302 10.1177/0748233714545502

[21] Al-Olayan E.M., El-Khadragy M.F., Metwally D.M. and Abdel Moneim A.E. (2014) Protective effects of pomegranate (Punica granatum) juice on testes against carbon tetrachloride intoxication in rats. BMC Complement. Altern. Med. 14, 164 10.1186/1472-6882-14-164 24884677 PMC4041339

[22] Sonmez M., Turk G., Ceribasi S., Ciftci M., Yuce A., Guvenc M. et al. (2014) Quercetin attenuates carbon tetrachloride-induced testicular damage in rats. Andrologia 46, 848–858 10.1111/and.12159 24020584

[23] Alpers D.H. and Isselbacher K.J. (1968) Biochemical effects of CCl4 on rat intestinal mucosa. Biochim. Biophys. Acta Gen. Subj. 158, 414–424 10.1016/0304-4165(68)90295-X 5665253

[24] Foaud M.A., Kamel A.H. and Abd El-Monem D.D. (2018) The protective effect of N-acetyl cysteine against carbon tetrachloride toxicity in rats. J. Basic Appl. Zool. 79, 14 10.1186/s41936-018-0022-x

[25] Doumas B.T. (1975) Standards for total serum protein assays–a collaborative study. Clin. Chem. 21, 1159–1166 10.1093/clinchem/21.8.1159 1169135

[26] Ohkawa H., Ohishi N. and Yagi K. (1979) Assay for lipid peroxides in animal tissues by thiobarbituric acid reaction. Anal. Biochem. 95, 351–358 10.1016/0003-2697(79)90738-3 36810

[27] Ellman G.L. (1959) Tissue sulfhydryl groups. Arch. Biochem. Biophys. 82, 70–77 10.1016/0003-9861(59)90090-6 13650640

[28] Engvall E. and Perlmann P. (1971) Enzyme-linked immunosorbent assay (ELISA). Quantitative assay of immunoglobulin G. Immunochemistry 8, 871–874 10.1016/0019-2791(71)90454-X 5135623

[29] Bonomini M., Dottori S., Amoroso L., Arduini A. and Sirolli V. (2004) Increased platelet phosphatidylserine exposure and caspase activation in chronic uremia. J. Thromb. Haemost. 2, 1275–1281 10.1111/j.1538-7836.2004.00837.x 15304031

[30] Dutta S., Chakraborty A.K., Dey P., Kar P., Guha P., Sen S. et al. (2018) Amelioration of CCl4 induced liver injury in swiss albino mice by antioxidant rich leaf extract of Croton bonplandianus Baill. PLoS ONE 13, e0196411 10.1371/journal.pone.0196411 29709010 PMC5927454

[31] Sallam A.A.A., El-Massry S.A. and Nasr I.N. (2009) Chemical analysis of mucus from certain land snails under Egyptian conditions. Arch. Phytopathol. Plant Prot. 42, 874–881 10.1080/03235400701494448

[32] El-Zawawy N.A. and Mona M.M. (2021) Antimicrobial efficacy of Egyptian Eremina desertorum and Helix aspersa snail mucus with a novel approach to their anti-inflammatory and wound healing potencies. Sci. Rep. 11, 24317 10.1038/s41598-021-03664-3 34934098 PMC8692597

[33] Grisham M.B. and Granger D.N. (1988) Neutrophil-mediated mucosal injury. Role of reactive oxygen metabolites. Dig. Dis. Sci. 33, 6S–15S 10.1007/BF01538126 2831016

[34] Unsal V., Cicek M. and Sabancilar I. (2021) Toxicity of carbon tetrachloride, free radicals and role of antioxidants. Rev. Environ. Health 36, 279–295 10.1515/reveh-2020-0048 32970608

[35] Pena-Llopis S., Pena J.B., Sancho E., Fernandez-Vega C. and Ferrando M.D. (2001) Glutathione-dependent resistance of the European eel Anguilla anguilla to the herbicide molinate. Chemosphere 45, 671–681 10.1016/S0045-6535(00)00500-2 11680763

[36] Safhi M.M. (2018) Nephroprotective effect of Zingerone against CCl4-induced renal toxicity in Swiss albino mice: molecular mechanism. Oxid Med. Cell Longev. 2018, Article ID 2474831 10.1155/2018/2474831 29636837 PMC5831687

[37] Wang M., Niu J., Ou L., Deng B., Wang Y. and Li S. (2019) Zerumbone protects against carbon tetrachloride (CCl4)-induced acute liver injury in mice via inhibiting oxidative stress and the inflammatory response: involving the TLR4/NF-kappaB/COX-2 pathway. Molecules 24, 1964 10.3390/molecules24101964 PMC6571963 31121820

[38] Wang C., Ma C., Fu K., Gong L.H., Zhang Y.F., Zhou H.L. et al. (2021) Phillygenin attenuates carbon tetrachloride-induced liver fibrosis via modulating inflammation and gut microbiota. Front Pharm. 12, 756924 10.3389/fphar.2021.756924 PMC8490881 34621179

